# Soil microbial community structure is unaltered by plant invasion, vegetation clipping, and nitrogen fertilization in experimental semi-arid grasslands

**DOI:** 10.3389/fmicb.2015.00466

**Published:** 2015-05-20

**Authors:** Chelsea J. Carey, J. Michael Beman, Valerie T. Eviner, Carolyn M. Malmstrom, Stephen C. Hart

**Affiliations:** ^1^Department of Plant Pathology and Microbiology, University of California, MercedMerced, CA, USA; ^2^Life and Environmental Sciences, University of California, MercedMerced, CA, USA; ^3^Sierra Nevada Research Institute, University of California, MercedMerced, CA, USA; ^4^Department of Plant Sciences, University of California, DavisDavis, CA, USA; ^5^Department of Plant Biology, Michigan State UniversityEast Lansing, MI, USA

**Keywords:** clipping, environmental change, invasive species, Mediterranean, microbial community structure, nitrogen fertilization, soil, stability

## Abstract

Global and regional environmental changes often co-occur, creating complex gradients of disturbance on the landscape. Soil microbial communities are an important component of ecosystem response to environmental change, yet little is known about how microbial structure and function respond to multiple disturbances, or whether multiple environmental changes lead to unanticipated interactive effects. Our study used experimental semi-arid grassland plots in a Mediterranean-climate to determine how soil microbial communities in a seasonally variable ecosystem respond to one, two, or three simultaneous environmental changes: exotic plant invasion, plant invasion + vegetation clipping (to simulate common management practices like mowing or livestock grazing), plant invasion + nitrogen (N) fertilization, and plant invasion + clipping + N fertilization. We examined microbial community structure 5–6 years after plot establishment via sequencing of >1 million 16S rRNA genes. Abiotic soil properties (soil moisture, temperature, pH, and inorganic N) and microbial functioning (nitrification and denitrification potentials) were also measured and showed treatment-induced shifts, including altered NO^−^_3_ availability, temperature, and nitrification potential. Despite these changes, bacterial and archaeal communities showed little variation in composition and diversity across treatments. Even communities in plots exposed to three interacting environmental changes were similar to those in restored native grassland plots. Historical exposure to large seasonal and inter-annual variations in key soil properties, in addition to prior site cultivation, may select for a functionally plastic or largely dormant microbial community, resulting in a microbial community that is structurally robust to single and multiple environmental changes.

## Introduction

Positioned at the interface of the plant-soil-atmosphere system, soil microbial communities are hypothesized to play important roles in mediating ecosystem response to anthropogenic environmental change (Swift et al., 1998; Talbot et al., 2008; Zak et al., 2011). This hypothesis rests upon at least two ideas. The first is the well-documented truism that soil microorganisms transform nitrogen (Davidson et al., 1990; Schimel and Bennett, 2004), consume and produce critical trace gasses (e.g., CO_2_, CH_4_, N_2_O, Conrad, 1996), and contribute to the carbon (C) storage capacity of ecosystems (van der Heijden et al., 2008; Schimel and Schaeffer, 2012). The second idea, which this study examines, is that biotic and abiotic factors, such as plant composition and soil moisture and temperature, influence microbial-mediated processes by either directly affecting the activity of resident microorganisms or by selecting for compositionally and functionally distinct microbial communities (Schimel and Gulledge, 1998; Bissett et al., 2013). Although soil microbial structure (composition and diversity) often shifts in response to changes in biotic and abiotic variables (Frey et al., 2004; Zhang et al., 2005; Ingram et al., 2008; Lesaulnier et al., 2008; Campbell et al., 2010; Ramirez et al., 2012; Coolon et al., 2013; Philippot et al., 2013), this does not universally occur (Waldrop and Firestone, 2006; Lamb et al., 2011; Marshall et al., 2011; Hagedorn et al., 2013). Critically, relatively little is known about how soil microbial communities respond to multiple co-occurring environmental factors in ecosystems that experience strong seasonal variability in temperature and precipitation, such as Mediterranean-type semi-arid grasslands (Docherty et al., 2012).

Many semi-arid grasslands located in Mediterranean climates are simultaneously impacted by multiple environmental changes. This partly stems from their role in sustaining livestock and croplands (Lavorel et al., 1998), and also from their close proximity to urban environments that contribute significantly to regional N emissions and act as ports of entry for exotic species (Vitousek et al., 1997; Fenn et al., 2003; Säumel and Kowarik, 2010). As a result, three factors that frequently affect these ecosystems are exotic plant invasion, elevated N deposition, and vegetation removal (Sala et al., 2000). Vegetation removal—while perhaps not thought of as a global or regional change itself—occurs to a large extent in grasslands as a result of land management practices such as livestock grazing and mowing (DiTomaso et al., 2007). It thus represents a potentially significant factor that may be of consequence for current and future ecosystems (Wardle and Bardgett, 2004).

Exotic plant invasion, elevated N deposition, and vegetation removal all have the potential to influence soil properties and subsequently microbial structure and function. Exotic plants (here defined as non-native species that have been introduced to a novel range) can redistribute nutrients within the plant-soil continuum, alter the quantity and quality of litter inputs in the soil, and influence soil temperature and moisture (Ehrenfeld, 2010). However, the direction and magnitude of these changes are variable (reviewed in Liao et al., 2008; Vilà et al., 2011), as are the reported effects of exotic plants on soil microbial communities (Batten et al., 2006; Xiao et al., 2014). For example, many exotic plants increase N availability, rates of N cycling, and the abundances of specific N-cycling microorganisms such as ammonia oxidizers (Hawkes et al., 2005; Liao et al., 2008; Vilà et al., 2011; Piper et al., 2015). Others, however, decrease N availability and rates of N cycling (Dassonville et al., 2011). For instance, *Aegilops triuncialis* and *Elymus caput-medusae*—two exotic species invading the western United States—have been shown to reduce litter decomposition (Bovey et al., 1961; Drenovsky and Batten, 2007), and in the case of *A. triuncialis*, this resulted in lower soil total N compared to uninvaded soils (Drenovsky and Batten, 2007).

Moreover, elevated N supply resulting from direct fertilization or atmospheric deposition can alter plant community composition, increase net primary production (NPP) and soil N availability, and reduce soil pH (Fenn et al., 2003, 2010). Such changes may alter the relative abundances of putative copiotrophic taxa (“r-selected;” e.g., *Actinobacteria, Bacteriodetes*, and β-*Proteobacteria*), oligotrophic taxa (“K-selected;” e.g., *Acidobacteria* and *Verrucomicrobia*, Fierer et al., 2007, 2012; Ramirez et al., 2012), and specific N-cycling microorganisms. For instance, ammonia-oxidizing bacteria (AOB) may respond more strongly than ammonia-oxidizing archaea (AOA; Di et al., 2010) to increases in NH^+^_4_ availability, resulting in higher AOB/AOA ratios (Shen et al., 2011).

By reducing transpiration and organic matter input into the soil, vegetation removal can increase soil moisture and temperature while decreasing nutrient and C pools (Wan et al., 2002; Wan and Luo, 2003; Ingram et al., 2008). Although long-term vegetation removal studies that monitor microbes are scarce, lower substrate availability associated with the removal of organic matter may reduce rates of N cycling and increase the ratio of oligotrophic to copiotrophic taxa (Fierer et al., 2007).

In this study, we examined the extent to which exotic plant invasion, elevated N supply, and removal of aboveground vegetation drive changes in microbial function and community structure in an ecosystem shaped by strong seasonal variability. We sampled from a long-term field experiment 5–6 years after establishment in Mediterranean-climate California, which is characterized by hot, dry summers and cool, wet winters. To identify potential controlling mechanisms on microbial community composition and links between microbial structure and function, we measured abiotic soil properties (e.g., soil moisture, temperature), rates of microbial-mediated processes (e.g., N cycling), and microbial community composition and diversity (using Illumina sequencing). Treatments were combined in a partial factorial design, where plant communities were either seeded with native species only or with native and exotic species, including the two exotics *A. triuncialis* and *E. caput-medusae*. The invaded plant communities were then subjected to a factorial combination of vegetation clipping and N fertilization. We tested the hypothesis that simulated environmental change in Mediterranean-climate systems will alter microbial-mediated processes through selection for distinct microbial communities. Specifically, we predicted that potential rates of nitrification and denitrification would decrease with exotic plant invasion and vegetation clipping, and that concurrent shifts in overall microbial structure would be associated with decreases in the relative abundances of putative copiotrophic and N cycling taxa. We predicted that the opposite trends would be observed with N fertilization, and that—when combined—vegetation clipping would moderate the impacts of N fertilization.

## Methods

### Experimental design and sampling

This study was conducted at an experimental site located in Davis, California, USA (38°32′45.52″N, 121°47′05.37″W). The site was cultivated for agricultural purposes prior to 1985, and then left fallow until the establishment of this experiment. Vegetation treatments were seeded at the beginning of the 2007–08 growing season; clipping and removal of vegetation was initiated at the end of the first growing season, while fertilization was initiated during the 2008–09 growing season. Soils were dominated by the Reiff series (75%, coarse-loamy, mixed, superactive nonacid, thermic Mollic Xerofluvents) and to a lesser extent the Brentwood soil series (25%, fine, smectitic, thermic Typic Haploxerepts) with a 0–2% slope (USDA Web Soil Survey, http://websoilsurvey.sc.egov.usda.gov). This site experiences a Mediterranean-type climate characterized by hot dry summers and cool wet winters, with a mean annual air temperature of 15.7°C and mean annual precipitation of 485 mm.

We sampled from five treatments, each of which had eight replicates arranged in a randomized complete block design: native, invaded, invaded + clipped, invaded + N fertilized, invaded + clipped + N fertilized. The native treatment was seeded with plants native to California grasslands: *Bromus carinatus, Elymus glaucus, Elymus triticoides, Acmispon americanus, Lupinus bicolor, Stipa pulchra, Poa secunda*, and *Festuca microstachys*. The invaded treatment was seeded with native, naturalized exotic, and invasive exotic plants: native–same species as listed above; naturalized–*Avena fatua, Bromus hordeaceus, Festuca perennis*, and *Trifolium subterraneum*; invasive–*A. triuncialis* and *E. caput-medusae*. Naturalized exotic plants were defined as those that have been present in California grasslands since the mid to late 1800s and which are now ubiquitous on the landscape; invasive exotic plants were defined as those that are currently spreading throughout California and which pose significant threats to ecosystem structure and function (California Invasive Plant Council Inventory, http://www.cal-ipc.org/paf/).

Plant communities were established by adding 139 g of seed to each 1.5 × 1.5 m plot. In the native treatment, species were planted at an equal proportion by seed weight. The invaded treatment was seeded with equal parts natives, naturalized, and invasive species (by seed weight). Within each group there was an equal proportion of the component species. To exhaust the existing seed bank, the site was irrigated prior to seeding, and plants that subsequently germinated were treated with glyphosate. Non-planted species were weeded periodically, but seeded species were allowed to vary in abundance. We used a modified Daubenmire approach to determine the species percent cover during the spring of each year, at the time of peak flowering (of the later-season species). Based on these data, native and invaded treatments were distinct in plant composition from the time of treatment establishment through 2012, with native treatments dominated by native species and invaded treatments dominated by exotic species (Table S1 in Supplementary Material). In 2013, native and invaded treatments had more moderate distinctions; native, naturalized, and invasive cover of native plots averaged 43, 35, and 4%, respectively; native, naturalized, and invasive cover of invaded plots averaged 34, 22, and 9%, respectively (see Table S2 in Supplementary Material for details on % cover of individual species during 2013).

Fertilization and clipping were conducted on invaded plant communities only, because this mixed community type is representative of grassland composition today. Fertilized treatments received two to three applications of aqueous N fertilizer annually, totaling a rate of 45 kg NH_4_NO_3_-N ha^−1^ y^−1^. This level of N supply is nearly 10 fold greater than background deposition rates at our site, but is well within the range of current deposition rates found elsewhere in California (Fenn et al., 2010). Each spring, biomass was cut at a height of 5 cm and removed in order to simulate common management techniques such as livestock grazing or mowing. During the 2012–13 growing season, fertilizer was applied on December 4, 2012, February 20, 2013, and March 26, 2013; plots were clipped on May 2, 2013.

We collected soils near the time of peak biomass in April of 2013, 6 years after establishment of plant communities and spring clipping, and after 5 years of fertilization. Each treatment had eight replicate soil cores, where one replicate was a composite of five soil cores (1.9 cm diameter × 15 cm deep), each taken at random locations from within a 1.5 × 1.5 m^2^ plot. This sampling strategy provided us with a good estimate of the mean soil properties of the plot, which was our unit of replication. Soil was sieved field-moist (2 mm) and subsamples of the composited soils were stored at −20°C for molecular analysis. The rest of the soil was stored at 4°C for measurement of soil properties and microbial functioning.

### Abiotic soil characteristics

To determine the effects of treatment on soil properties, soils were analyzed for gravimetric water content (GWC), pH, total C and N, and inorganic N availability. In addition, *in situ* measurements of soil volumetric water content (VWC) and temperature were also collected. GWC was assessed by drying field moist soil at 105°C to a constant mass. VWC was measured using Time Domain Reflectometer probes (TDR; MiniTrase, Soil Moisture Equipment Corp., Santa Barbara, CA, USA) that were installed vertically into the soil (15 cm depth) in October 2011 (six replicates per treatment). Temperature was measured using HOBO dataloggers (Onset Corporation, Cape Cod, MA, USA), which we placed at 7.5 cm depth (one sensor/plot, eight replicates per treatment). Soil pH was measured (using a pH combination electrode) on a 1:2 (w/v) soil to 0.01 M CaCl_2_ suspension that had equilibrated for 30 min (Orion DUAL STAR meter, Thermo Scientific, Waltham, MA, USA). We used ground, oven dried soil to analyze total C and N on an Elemental Combustion System (Costech Analytical Technologies Inc., Valencia, CA, USA). Total C and N were not expected to vary greatly within 1 year, since the annual flux in this pool is much smaller than the total pool size (Binkley and Hart, 1989; Eviner and Firestone, 2007); thus, we composited samples that were taken in the same way as described above from four dates in 2012–13 (November, January, April [corresponding to when soils were collected for microbial analysis in this study], and July) prior to analysis.

To gauge how soil inorganic N responded to each treatment, we paired measurements of instantaneous pools of NH^+^_4_ and NO^−^_3_ with *in situ* measurements of NH^+^_4_ and NO^−^_3_ availability using ion-exchange resin bags (Binkley and Matson, 1983; Binkley, 1984). Pools of NH^+^_4_ and NO^−^_3_ were measured by extracting 15 g of field-moist soil with 100 mL of 2 M KCl. Soil suspensions were shaken for 1 h on a reciprocating shaker, filtered with Whatman No. 1 filter paper pre-leached with deionized water, and stored at −20°C until analysis on a Lachat AE Flow Injection Autoanalyzer (Lachat Instruments, Inc., Milwaukee, WI, USA). Ion-exchange resin bags were made by weighing15 mL of cation and anion resin beads (J.T. Baker Mixed Bed Exchange Resin, IONAC NM-60 H^+^/OH^−^ Form, Type 1, 16–50 Mesh) into nylon stockings, which were subsequently tied shut, and placed in the soil at 7.5 cm depth on February 26, 2013 (1 bag/plot; 12 replicates per treatment). The resin bags were removed from the field approximately 6 weeks later when soil samples were collected for molecular analysis. After air drying, the resin bags were suspended in 50 mL of 2M KCl, shaken uncovered for 1 h on an orbital shaker (180 rpm), and filtered using pre-leached Whatman No. 1 filter paper. The resin filtrates were stored at −20°C until analysis on a Lachat AE Flow Injection Autoanalyzer.

### Microbial functioning

Microbial functioning was assessed in the laboratory as potential rates of nitrification and denitrification. We estimated potential rates of nitrification using the shaken soil-slurry method (Hart et al., 1994), a method used to approximate the *in situ* activity of nitrifying enzymes in soil. Briefly, we added 15 g of field-moist soil to 100 mL NH^+^_4_ and PO^3−^_4_ solution (1.5 mM of NH^+^_4_ and 1 mM of PO^3−^_4_, pH = 7.2) in a 250-mL flask and capped with a rubber stopper (containing a hole to allow gas exchange but minimize water loss). Flasks were placed on an orbital shaker (180 rpm) for 24 h. At 2, 4, 22, and 24 h, 10 mL of suspension was removed from each flask and centrifuged at 8000 ×*g* for 8 min. The supernatant (5 ml) was removed from the centrifuged soil slurry, placed into a disposable polypropylene tube, capped, and stored at −20°C until analysis for NO^−^_3_.

Potential denitrification, which measures the *in situ* denitrifying enzyme activity of soils, was assessed using the protocol described by Smith and Tiedje (1979). Here, 50 g of field-moist soil was combined with NO^−^_3_ and labile C (0.1 mg N-NO^−^_3_ g^−1^ soil, 1 mg C-glucose g^−1^ soil, and 1 mg C-glutamic acid g soil^−1^) in a 250 mL flask. The flask was sealed tight with a rubber stopper fitted with an airtight septum, and was alternately evacuated (3 min) and flushed with N_2_ (1 min) three times to create anaerobic conditions. We then injected 20 mL of acetylene gas into each flask in order to inhibit the reduction of N_2_O to N_2_. The soils were incubated for 90 min on an orbital shaker (180 rpm) and sampled at 30 and 90 min by removing 15 mL of the headspace. Gas samples were stored in evacuated Exetainer® vials for 2 weeks prior to analysis for N_2_O production on a Shimadzu GC-2014 electron capture detector (Shimadzu Corporation, Columbia, MD, USA).

### DNA extraction, quantification, and barcoded amplicon sequencing on the illumina MiSeq platform

To determine treatment effects on soil microbial community structure, microbial DNA was extracted from 0.25 g of soil (±0.5 g) using a MO BIO PowerSoil DNA Isolation Kit (MO BIO Laboratories Inc., Carlsbad, CA, USA) following the manufacturer's instructions. Extracted DNA was quantified using a Quant-iT PicoGreen ds DNA Assay Kit (Life Technologies, Carlsbad, CA, USA) and diluted with sterile water to 1 ng uL^−1^ DNA. DNA extracts (10 μL per sample) were shipped overnight on dry ice to Argonne National Laboratory (Lemont, Illinois, USA) for amplicon library preparation of the V4 region of the 16S rRNA gene (515f and 806r primers; Caporaso et al., 2011, 2012), following the Earth Microbiome Project protocol (Gilbert et al., 2010). Amplicons were subsequently sequenced using the Illumina MiSeq2000 platform.

### Sequence analysis

We used default parameters in Quantitative Insights into Microbial Ecology (QIIME, Caporaso et al., 2010) for quality control. Specifically, reads were excluded if there were more than three consecutive low-quality base calls, if less than 75% of the read length was consecutive high-quality base calls, if a Phred score was below three, if one or more ambiguous calls were present, or if the length was less than 75 bases (Bokulich et al., 2013). After forward and reverse reads were joined and demultiplexed, we picked operational taxonomic units (OTU) at 97% similarity using open reference UCLUST against the 13_8 release of the Greengenes database. Reads that did not match any sequences in the database were clustered *de novo*.

Sequencing of 40 samples resulted in approximately 2.1 million reads in total, 908,200 of which were removed due to the presence of ambiguous bases in the sequences. We found that the vast majority of these were due to an “N” located three base pairs from the beginning of the sequence. We consequently processed the sequences in two ways: (1) with a conservative approach that adhered to the quality control procedures above, despite the loss of sequences; and (2) by trimming all sequences by 3 bp from the start position and then processing them in the same fashion. We found no difference in our ultimate conclusions—i.e., there was no bias across treatments, replicates, and/or particular microbial groups—and report results from the conservative approach here. After quality control, 1.1 million reads remained, averaging 29,441 reads per sample. Sequences have been submitted to the NCBI Sequence Read Archive (Accession number SRR1980669).

### Statistical analyses

To determine main and interactive treatment effects on soil abiotic characteristics and microbial functioning, we used R statistical software (R Core Team, 2014) to perform One- and Two-Way analysis of variance (ANOVA). One-Way ANOVA was used to determine differences among native and invaded plant communities, while Two-Way ANOVA determined the main effects of clipping (unclipped or clipped) and fertilization (unfertilized or fertilized), and clipping × fertilization interactions. In all models, block was included as a random factor.

In QIIME, sequences were subsampled to an even depth of 12,000 reads prior to estimating relative taxon abundances and running diversity analyses. Alpha diversity was measured using Shannon diversity index, Chao1 richness, Faith's phylogenetic diversity, and Simpson's diversity; treatment differences among these metrics were assessed using One- and Two-Way ANOVA as described above. Beta diversity was visualized in the R phyloseq package (McMurdie and Holmes, 2013) using principal coordinate analysis (PCoA) from unweighted and weighted Unifrac distances, which uses the overlap in branch lengths to estimate phylogenetic distance between pairs of bacterial communities (Lozupone and Knight, 2005). Since results were qualitatively similar between the unweighted and weighted Unifrac distances (in regards to the effect of treatments and patterns of within and between treatment variation; see Figures S1, S2 in Supplementary Material), our discussion focuses on the weighted Unifrac metric. The multivariate permutation tests perMANOVA was used to determine if beta diversity differed significantly among treatments (permutations = 999). In addition to running diversity analyses that included all taxa, we ran separate beta diversity analyses and permutation tests for a subset of taxa that included only nitrifying microorganisms (*Thaumarchaeota, Nitrospira, Nitrosomonadales*); this allowed us to determine whether nitrifying microbial communities differed significantly across treatments. Results were visualized using PCoA, in the same way as described above. Finally, One- and Two-Way ANOVA were used to determine main and interactive treatment effects on the relative abundances of select taxa. To account for multiple comparisons, we assessed treatment effects with and without using a sequential Bonferroni correction at the significance level (*P* < 0.05; Holm, 1979; Moran, 2003). Treatment effect sizes for relative abundances of microbial taxa were calculated as % effect = (treatment – control)/control, where the control for invaded plant communities was the native plant community and the control for all other treatments was the invaded plant community.

We used Mantel tests to relate variations in microbial community composition across treatments with soil characteristics; this was done by correlating pairwise weighted UniFrac distances with Euclidean distances of soil NH^+^_4_ and NO^−^_3_ concentrations, total C and N, moisture, temperature, and pH (Jones et al., 2009). Mantel tests were also used to compare community composition with nitrification and denitrification potentials. In addition, diversity metrics and the relative abundances of the most abundant phyla were correlated with soil characteristics using Pearson correlations, which were visualized using the corrplot package in R (Wei, 2013).

## Results and discussion

### Effects of invasion, clipping, and fertilization on soil properties and microbial functions

**Figure 1:**
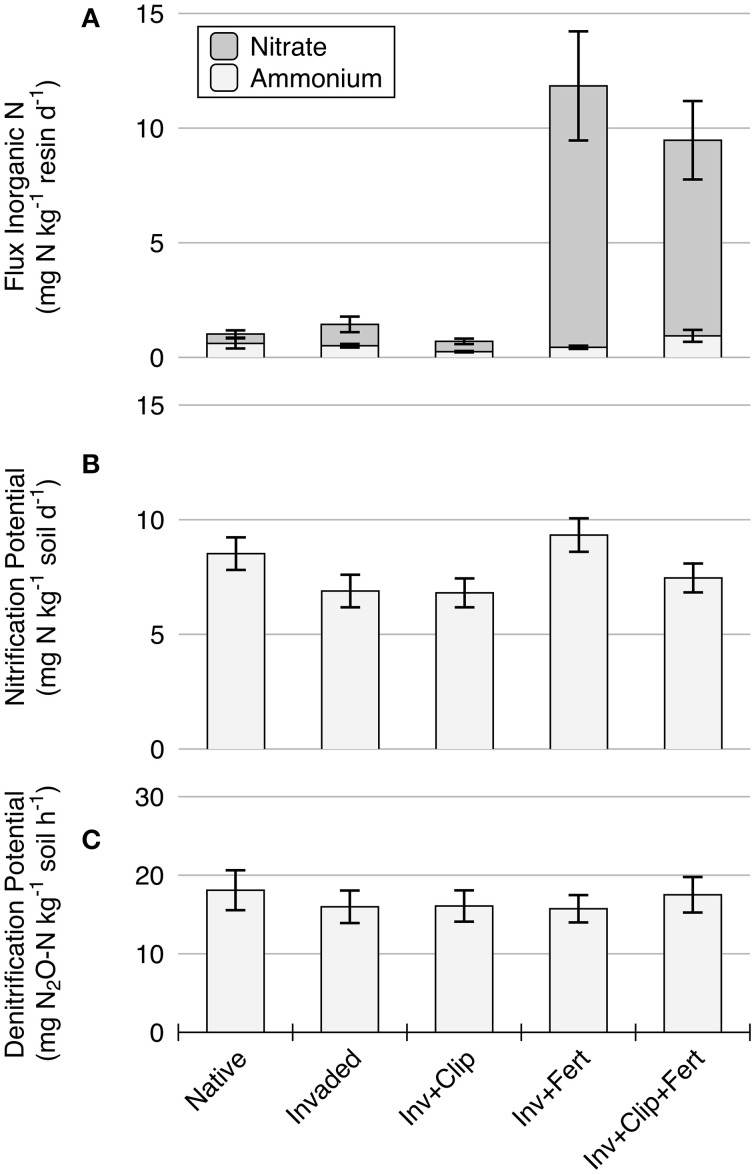
**Treatment effects on (A) time-integrated measurements of NO^−^_3_ and NH^+^_4_ as measured by ion-exchange resin bags, (B) nitrification potential, and (C) denitrification potential**. Bars = mean ± 1 standard error.

We observed treatment-induced changes in some key soil properties, including soil temperature, pH, NO^−^_3_ availability and total C and N (Table 1). Invaded plant communities, for example, had lower soil total C and N than native plant communities, while clipping reduced NO^−^_3_ concentrations and increased maximum daily soil temperature. In addition, fertilization dramatically increased NO^−^_3_ availability (Figure 1) and slightly reduced soil pH compared to the unfertilized control. These results confirm that each treatment influenced aspects of the soil environment in ways that are consistent with what we would expect based on prior studies (Wan et al., 2002; Fenn et al., 2003; Drenovsky and Batten, 2007).

**Table 1:** **(A) Mean values of abiotic soil characteristics for the five different treatments from 0 to 15 cm soil depth. Parentheses denote standard errors of the mean** (***n*** **= 8). (B)** ***P***-**values associated with treatment differences**.

	**VWC (%)**	**GWC (%)**	**Total *N* (g N kg^−1^ soil)**	**Total *C* (g N kg^−1^ soil)**	**C/N**	**pH**	**Max Temp (°C)**	**Min Temp (°C)**	**[NH^+^_4_] (mg N kg^−1^ soil)**	**[NO^−^_3_] (mg N kg^−1^ soil)**
**(A) TREATMENTS**										
Native	21.86 (1.73)	16.17 (0.32)	1.24 (0.03)	12.20 (0.29)	9.80 (0.08)	6.37 (0.03)	17.45 (0.93)	13.11 (0.25)	0.13 (0.02)	1.56 (0.18)
Invaded	20.78 (1.89)	17.60 (0.38)	1.21 (0.02)	11.83 (0.26)	9.79 (0.1)	6.40 (0.04)	16.82 (0.59)	12.92 (0.11)	0.15 (0.03)	1.46 (0.19)
Inv + Clip	22.23 (1.99)	16.55 (0.74)	1.23 (0.04)	12.25 (0.49)	9.94 (0.1)	6.40 (0.04)	19.87 (0.84)	12.89 (0.23)	0.18 (0.04)	0.89 (0.15)
Inv + Fert	18.52 (1.01)	16.60 (0.69)	1.24 (0.02)	12.24 (0.27)	9.87 (0.13)	6.36 (0.03)	17.32 (0.40)	13.10 (0.15)	0.10 (0.01)	2.00 (0.23)
Inv + Clip + Fert	17.00 (1.48)	18.15 (1.38)	1.22 (0.03)	12.03 (0.38)	9.83 (0.13)	6.31 (0.04)	18.06 (0.52)	13.26 (0.08)	0.14 (0.02)	1.23 (0.11)
**(B) ANOVA *P*-VALUES**										
Invasion	0.76	0.08	**0.04**	**0.04**	0.89	0.33	0.46	0.29	0.99	0.97
Clipping	0.99	0.79	0.93	0.66	0.61	0.42	**0.00**	0.56	0.36	**0.00**
Fertilization	**0.04**	0.48	0.48	0.67	0.94	**0.02**	0.27	**0.05**	0.56	0.12
Clipping x Fertilization	0.39	0.21	0.24	0.18	0.36	0.49	**0.02**	0.51	0.78	0.95

*VWC, volumetric water content; GWC, gravimetric water content; Max and Min Temp, maximum and minimum daily soil temperature at 7.5 cm depth. P-values associated with invasion were generated by comparing native and invaded communities using a One-Way ANOVA, and P-values for clipping and fertilization were generated using a Two-Way ANOVA. Significant (P < 0.05) and nearly significant (P < 0.1) values are highlighted in bold*.

Nitrification potential also varied by treatment, supporting our prediction that alterations in plant composition and abiotic variables can influence microbial functioning. Nitrification potential responded most significantly to nitrogen fertilization, with fertilized plots displaying higher rates than unfertilized plots (*P* = 0.05; Figure 1, Table S3 in Supplementary Material). This finding demonstrates an increase in nitrifying microbial activity with elevated substrate (NH^+^_4_) supply, and is congruent with prior studies that documented similar trends with fertilization (Mendum et al., 1999; Chen et al., 2014). A lack of NH^+^_4_ accumulation in fertilized soils provides evidence that *in situ* rates of nitrification, microbial immobilization, or plant uptake responded to elevated N supply as well (Table 1). In addition, nitrification potential demonstrated a weak trend between native and invaded plant communities, with lower rates in invaded vs. native treatments. This agrees with some previous reports on invasive plants (Corbin and D'Antonio, 2011; Dassonville et al., 2011), but not others (Hawkes et al., 2005; Parker and Schimel, 2010), and the trend was only nearly significant (*P* = 0.07; Figure 1, Table S3 in Supplementary Material). *A. triuncialis* and *E. caput-medusae*, two exotic species utilized in this study, have been shown to slow litter decomposition (Bovey et al., 1961; Drenovsky and Batten, 2007)—a process that releases inorganic N into the soil system (Chapman et al., 2006)—and could be one explanation for why nitrification rates were lower in the invaded treatment.

In contrast to potential rates of nitrification, denitrification potential did not significantly differ between any of the treatments (Figure 1). Denitrification is a facultative process catalyzed by a phylogenetically broad group of microorganisms under suboxic conditions (Hayatsu et al., 2008); the relative commonness of this process, along with the ability of denitrifiers to quickly synthesize enzymes in response to changing conditions (Rudaz et al., 1991) may result in high functional redundancy and functional stability (Bissett et al., 2013). Nitrification may be more sensitive to change, as this obligate process is carried out by a phylogenetically constrained group of ammonia oxidizing and nitrite oxidizing microorganisms (Hayatsu et al., 2008). As such, one explanation for our observed results is that “narrow” processes (e.g., nitrification) may be more responsive to environmental change than “broad” processes (e.g., denitrification, Schimel, 1995), which may have large scale implications for NH^+^_4_ and NO^−^_3_ availability and, by extension, N retention under changing scenarios.

### Effects of invasion, clipping, and fertilization on soil microbial community structure

**Figure 2:**
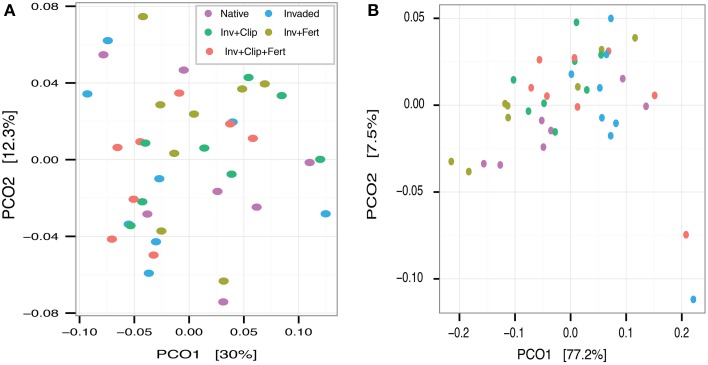
**Principal coordinates analysis based on the weighted UniFrac distance metric for (A) all taxa and (B) nitrifying taxa**. Together, the first two principle coordinates explained 42.3 and 84.7% of the variation in overall microbial community composition and composition of nitrifying taxa, respectively.

Despite observing changes in some soil abiotic variables and rates of nitrification across treatments, we found that bacterial and archaeal diversity and community composition were, for the most part, unchanged by exotic plant invasion, clipping, and N fertilization. Even the most heavily altered treatment harbored microbial communities that were similar to the native grassland treatment, contrasting with our overall hypothesis. Indeed, community richness, evenness, and phylogenetic diversity were statistically indistinguishable among treatments (Table 2). Principal coordinates analysis of the weighted UniFrac distance illustrated a lack of distinct clustering by treatment (Figure 2), which was confirmed using perMANOVA (Pseudo-F statistic = 1.01, *P* = 0.23). Similar findings were observed for nitrifying microorganisms when they were analyzed independent of the larger community (Figure 2; Pseudo-F statistic = 1.65, *P* = 0.15).

**Table 2:** **(A) Mean values of alpha diversity by treatment. Parentheses denote standard errors of the mean** (***n*** **= 8). (B)** ***P***-**values associated with treatment differences**.

	**Observed richness**	**Chao richness**	**Shannon diversity**	**Faith's phylogeny**	**Simpson's diversity**
**(A) TREATMENTS**					
Native	3973.1 (67.9)	7667.6 (238.6)	10.9 (0.07)	240.3 (3.7)	0.9983 (0.0)
Invaded	3927.3 (50.2)	7924.5 (324.1)	11.1 (0.07)	238.9 (2.6)	0.9985 (0.0)
Inv + Clip	3872.3 (141.7)	7808.1 (665.6)	10.9 (0.12)	234.3 (7.4)	0.9984 (0.0)
Inv + Fert	3911.9 (47.9)	8032.8 (273.3)	10.9 (0.07)	236.5 (3.1)	0.9984 (0.0)
Inv + Clip + Fert	3816.7 (118.9)	7518.3 (343.2)	10.8 (0.10)	232.1 (5.9)	0.9975 (0.0)
**(B) ANOVA *P*-VALUES**					
Invasion	0.56	0.33	0.55	0.32	0.32
Clipping	0.45	0.31	0.29	0.31	0.43
Fertilization	0.72	0.75	0.50	0.59	0.35
Clipping x Fertilization	0.84	0.71	0.62	0.76	0.39

*P-values associated with invasion were generated by comparing native and invaded communities using a One-Way ANOVA, and P-values for clipping and fertilization were generated using a Two-Way ANOVA. None of the alpha diversity metrics differed significantly by treatment (P < 0.05)*.

**Figure 3:**
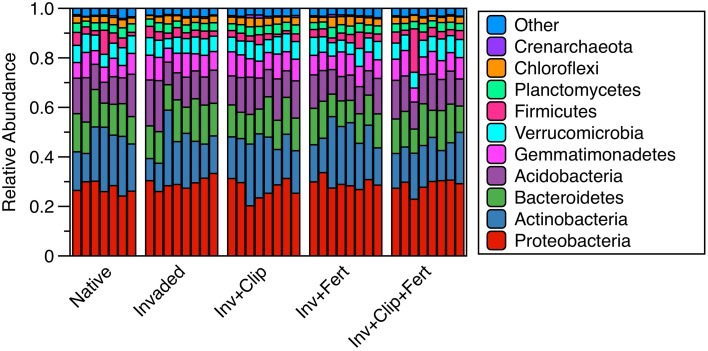
**Relative abundances (frequency) of the dominant phyla within and across treatments**. “Other” indicates the combined relative sequence abundance of the additional, rare phyla (28 phyla).

**Figure 4:**
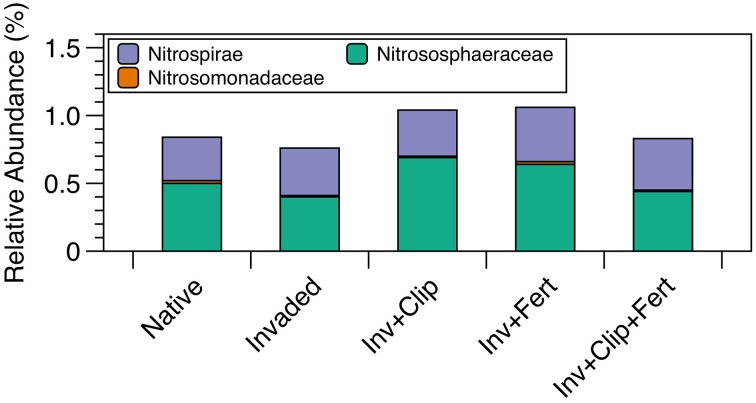
**Relative abundances (percent) of key taxa involved in nitrification**. Nitrosomondaceae is a family containing ammonia-oxidizing bacteria, Nitrososphaeraceaea is a family containing ammonia-oxidizing archaea, and Nitrospirae is a phylum containing nitrite-oxidizing bacteria.

The same phyla dominated all samples (Figure 3), with *Proteobacteria, Actinobacteria, Bacteriodetes, Acidobacteria*, and *Gemmatimonadetes* accounting for ca. 80% of sequences in each sample (range 80–82%). Twenty-six additional phyla were present in all soils but at lower abundances, and 10 other low-abundance phyla were present but not consistent across all soils (Figure S3 in Supplementary Material). However, there were a few key differences in the relative abundance of select taxa across treatments. In agreement with our predictions, *Nitrosomonadaceae* (a family containing AOB) were reduced in invaded relative to native plant communities (*P* < 0.05, Figure 4), and in clipped relative to unclipped treatments (nearly significant, *P* = 0.09). These trends, while not very strong, are congruent with the idea that, over time, invasion by certain exotic plants (e.g., *A. triuncialis* and *E. caput-medusae*) and yearly removal of aboveground vegetation can reduce C and N pools (Table 1, Drenovsky and Batten, 2007; Chen et al., 2014), potentially affecting substrate availability for specific N-cycling microorganisms. In addition to influencing the nutrient cycling capacity of the soil, there is some evidence that a shift in the ratio of oligotrophic to copiotrophic microorganisms can feedback to affect the resilience or resistance of a system (reviewed in de Vries and Shade, 2013). This is because copiotrophs are thought to be resilient to environmental change, while their oligotrophic counterpoints may be more resistant to change. While we observed small differences with clipping (reductions in *Proteobacteria* and increases in *Verrucomicrobia*, but *P* = 0.07 and *p* = 0.09, respectively; Table 3), in general the relative abundances of microbial groups—some of which can be generally identified as copiotrophic or oligotrophic—remained unaffected across each of these treatments.

**Table 3:** **Main and interactive effects of invasion, clipping, and N fertilization on (A) the 10 most abundant phyla and (B) nitrifying taxa. Effect sizes are presented for the relative changes in (A) and (B) by treatment**.

	**Invasion**		**Clipping**		**N fertilization**		**Clipping x N Fert**
	**Average % effect**	***P*-value**	**Average % effect**	***P*-value**	**Average % Effect**	***P*-value**	***P*-value**
**(A) DOMINANT PHYLA**							
Proteobacteria	+7.07	0.29	**−8.14**	**0.07**	+0.37	0.39	0.33
Actinobacteria	−10.67	0.36	+25.62	0.53	+25.51	0.93	**0.01**
Bacteriodetes	+9.31	0.70	−3.19	0.75	−10.67	0.59	0.14
Acidobacteria	+5.74	0.39	+7.17	0.36	−11.6	0.17	0.32
Gemmatimonadetes	+18.56	0.13	+4.33	0.64	−3.46	0.41	0.83
Verrucomicrobia	−5.93	0.35	**+7.94**	**0.09**	−0.65	0.73	0.35
Firmicutes	−1.37	0.25	−15.69	0.92	+14.13	0.13	0.17
Planctomyetes	+1.87	0.76	+8.74	0.99	+5.19	0.36	**0.05**
Chloroflexi	−2.77	0.59	+28.58	0.98	+25.96	0.97	0.00^*^
Crenarchaeota	−13.34	0.11	+112.84	0.42	+78.45	0.52	0.00^*^
**(B) NITRIFIERS**							
Nitrospirae	+32.09	0.37	−2.68	0.98	+4.54	0.17	0.53
Nitrososphaeraceaea	−13.34	0.11	+112.8	0.29	+78.45	0.69	0.00^*^
Nitrosomonadaceaea	**−45.54**	**0.05**	**−27.35**	**0.09**	+122.92	0.84	0.97

Main effects of invasion were determined by comparing native and invaded treatments using a One-Way ANOVA. Main and interactive effects of clipping and N fertilization were determined using a Two-Way ANOVA. Significant (P < 0.05) and nearly significant (P < 0.1) values are highlighted in bold. When sequential bonferroni corrected, only P-values with ^*^ were still significant. Main (single) factor effect sizes were calculated as = (treatment – control/control)^*^100, where the control for invasion was the native plant community and the control for clipping and N fertilization was the invaded plant community.

Many studies have focused on the effects of livestock grazing and herbivory on soil microbial communities (reviewed in Bardgett et al., 1998; Clegg, 2006; Ingram et al., 2008), but few have targeted the effects of aboveground vegetation removal on microbial community structure (Zhang et al., 2005; Jangid et al., 2011). Those that measured the influence of herbivory and livestock grazing have shown increases in rhizosphere microbial biomass and activity (as a result of elevated root exudation) and changes in microbial community composition (Bardgett et al., 1998; Clegg, 2006; Ingram et al., 2008). However, studies comparing grazed and ungrazed land cannot decouple the individual effects of vegetation removal, redistributed nutrient supply through animal waste, and soil compaction, so the role of vegetation removal is difficult to identify. Denef et al. (2009) mowed restored grasslands at different frequencies and reported that mowing frequency did not affect the relative abundances of PLFA biomarkers, findings that are similar to ours (Denef et al., 2009). If our results are indicative of a larger trend, microbial communities largely resist (or recover from) near complete vegetation removal during mowing or grazing of Mediterranean-type semi-arid grassland communities.

In contrast to our predictions and previous work (e.g., Marschner et al., 2003; Nemergut et al., 2008; Ramirez et al., 2010; Koyama et al., 2014), we found no significant effects of N fertilization on overall microbial community structure or the relative abundances of putative copiotrophic and N cycling taxa. One explanation for this is that many prior studies fertilized at higher rates for longer periods of time. However, Ramirez et al. (2010) found that microbial composition responded to low application rates (e.g., 34 kg N ha^−1^ y^−1^) in both a grassland and agricultural field, and that this response was stronger in treatments receiving higher amounts of N. In addition, Koyama et al. (2014) demonstrated significant structural shifts in the microbial community after 5 years of fertilizing with 10 kg N ha^−1^ y^−1^. Five years of fertilization with 45 kg N ha^−1^ y^−1^ as NH_4_NO_3_ therefore should have been sufficient to induce structural changes in the microbial community, yet we only observed changes in microbial activity in the form of nitrification potential.

Finally, although overall microbial community composition and diversity remained statistically unchanged by the interaction of clipping and N fertilization (Table 2, Figure 2), these treatments interacted to influence the relative abundances of *Actinobacteria, Planctomycetes, Chloroflexi*, and *Crenarchaeota* (Table 3), partially supporting our last prediction. The removal of aboveground vegetation can limit the recycling of nutrients and C through litter decomposition (Wan and Luo, 2003), and could explain why clipping decreased the positive response of these four phyla to fertilization. A recent study by Chen et al. (2014) found significant independent effects—but no interactions—of N fertilization and mowing on AOB and AOA gene copy numbers in a temperate steppe ecosystem; similar to this, we found nitrifying taxa to be insensitive to the interaction of these two treatments (Table 3), suggesting that the response of microbial groups to interacting environmental change will be complex and varied, with some taxa demonstrating higher sensitivity than others.

### Relationships between soil properties, microbial structure, and microbial functions

**Figure 5:**
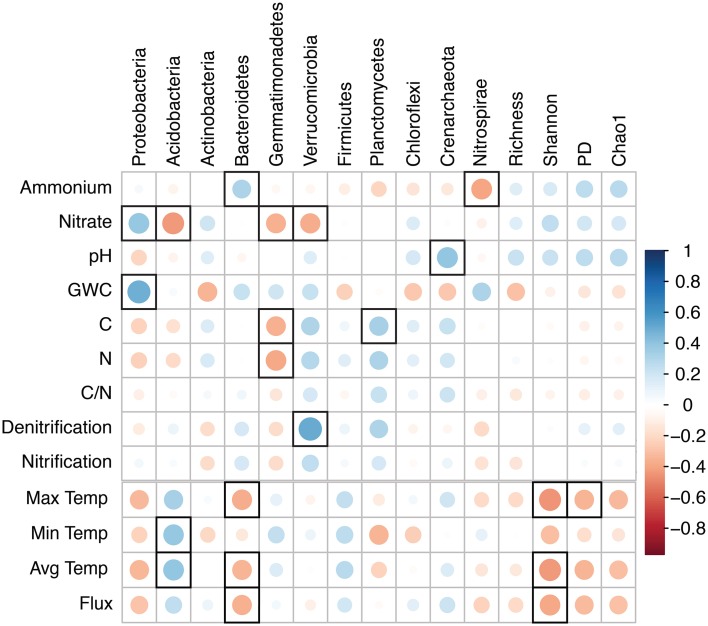
**Correlation matrix based on Pearson's Rank correlation coefficients between soil characteristics, potential nitrification and denitrification, the most abundant phyla, and alpha diversity metrics**. The size and intensity of color for each circle represents the strength of the correlation (the larger, darker circles demonstrate a strong correlation); blue colors illustrate positive correlations and red colors illustrate negative correlation coefficients. Correlations that are significant (*P* < 0.05) are encased in a bold black box. Ammonium and nitrate, pools expressed as mg N kg^−1^ soil; GWC, gravimetric water content; C, total C (g C kg^−1^ soil); N, total N (g N kg^−1^ soil); Max, Min, and Avg Temp, Maximum, Minimum, and Average daily soil temperature (°C at 7.5 cm soil depth); Flux, daily soil fluctuation in temperature (Max – Min); PD, Faith's phylogenetic diversity.

While microbial community structure remained relatively stable across treatments, several aspects of structure correlated with soil properties when collapsed across all samples (independent of treatment). Notably, Shannon and phylogenetic diversity demonstrated significant negative correlations with maximum daily soil temperature (Figure 5), and community composition (pairwise Unifrac distances) correlated with NH^+^_4_ concentrations and gravimetric soil moisture (Table 4). Community changes associated with increased NH^+^_4_ concentrations were driven in part by an increase in the relative abundance of *Bacteroidetes* (Figure 5). In the same way, changes in community composition with soil moisture were associated with shifts in the relative abundance of *Proteobacteria*. *Proteobacteria* were also positively correlated with NO^−^_3_ concentrations, while *Acidobacteria, Verrucomicrobia*, and *Gemmatimonadetes* displayed negative correlations with NO^−^_3_ availability. These correlations are consistent with the idea that the hyperdiverse phylum *Proteobacteria* may contain copiotrophic microorganisms, and that *Acidobacteria* and *Verrucomicrobia* may be oligotrophic (Fierer et al., 2007; Ramirez et al., 2012).

**Table 4:** **Relationship between soil properties, microbial functions, and microbial structure.Columns (A) and (B) list pearson correlation coefficients between soil properties and measurements of nitrification potential and denitrification potential, respectively. Column (C) lists Mantel correlation coefficients between weighted Unifrac distance, soil properties, and measurements of microbial function**.

	**(A) Nitrification potential**	**(B) Denitrification potential**	**(C) Weighted UniFrac distance**
[NH^+^_4_]	−0.14	0.11	**0.24**
[NO^−^_3_]	0.47^*^	0.03	0.13
Flux NH^+^_4_	0.02	0.14	0.07
Flux NO^−^_3_	**0.40**	0.22	−0.04
Total *C*	**0.42**	0.65^*^	−0.08
Total *N*	**0.37**	0.65^*^	−0.03
C:N	0.09	**0.36**	−0.05
Moisture	−0.07	−0.06	**0.27**
pH	−0.06	0.14	−0.04
Max temp	−0.33	−0.47^*^	0.07
Min temp	−0.17	−0.17	−0.02
Avg temp	−0.45^*^	−0.45^*^	0.08
Nitrification potential	–	0.51^*^	−0.01
Denitrification potential	0.51^*^	–	0.1

Moisture, gravimetric water content; max temp, maximum soil temperature at 7.5 cm depth, min temp, minimum soil temperature, avg temp, average soil temperature. Fluxes of NH^+^_4_ and NO^−^_3_ were measured using ion-exchange resin bags. Bold values indicate significance (P < 0.05). After sequential bonferroni corrected, only P-values with ^*^ were still significant.

The relationship between NO^−^_3_, *Proteobacteria*, and *Verrucomicrobia* likely explains why these two taxa were respectively reduced and elevated in clipped plots, which had lower NO^−^_3_ concentrations than unclipped plots. Although other abiotic variables (e.g., maximum temperature, [NH^+^_4_], pH) were affected by treatment, the magnitude of effect may not have been large enough to induce statistically significant changes in relative abundances at the phyla level. For instance, while Lauber et al. (2009) demonstrated significant trends in several dominant taxa across a gradient of pH from 4 to 9, fertilization at our site only reduced soil pH by 0.04, or an effect size of 0.01%. It is also possible that microbial community structure is more sensitive to those abiotic variables that did not change across treatments, such as soil C to N ratio (Marschner et al., 2003; Högberg et al., 2007).

Finally, we provide evidence that changes in microbial activity can occur independently of changes in microbial community composition, and so likely represent physiological responses to change. For example, the relative abundances of *Nitrosomonadaceae, Nitrososphaeraceae, and Nitrospirae*—individually and combined—failed to significantly explain observed changes in potential rates of nitrification. This could reflect differences in per cell nitrification rates (Jia and Conrad, 2009) or phenotypic changes in ammonia oxidizing microorganisms (Mendum et al., 1999). When compared across all treatments, neither nitrification nor denitrification potential correlated with microbial community composition (Table 4). These findings indicate that RNA-based analyses of microbial activity (i.e., transcriptomics, DeAngelis and Firestone, 2012), will be an important component of future studies, as microbial functioning may be sensitive to environmental change, even if community structure is relatively stable.

### Sources of microbial stability

Altogether our results suggest that microbial communities in semi-arid Mediterranean-type grasslands can remain structurally stable even when subjected to multiple environmental changes. This observed stability may be attributed to several factors, and has implications for understanding and modeling the response of microbial communities to global and regional change. First, Mediterranean climates are marked by strong seasonal and interannual changes in soil N availability (Parker and Schimel, 2011), plant composition (Gutknecht et al., 2012), temperature, and especially moisture—changes that are often large compared to those observed between treatments (Carey, 2014). For instance, with little to no precipitation during the summer months, soil moisture levels at our site drop from an average 20% (VWC) during the growing season (with a peak of around 30%) to 5% in June through August (Carey, 2014). Microorganisms within this environment should be adapted to cope with significant changes in soil variables—for example, through individual metabolic capabilities, gene expression, mixotrophy, dormancy, and acquisition of new genes (Ochman et al., 2000; Fraser et al., 2007; Lennon and Jones, 2011; Shade et al., 2012). As such, experimental treatments may not be sufficient to push soil microbial communities outside the boundaries of historical environmental variation where compositional shifts are likely to occur (Waldrop and Firestone, 2006). This idea, supported by our study and others (Waldrop and Firestone, 2006; Cruz-Martínez et al., 2009; Curiel Yuste et al., 2014), challenges the notion that, as a climatic transitional region, Mediterranean ecosystems will be hypersensitive to global and regional change (Lavorel et al., 1998).

Whether a microbial community shifts in composition may also be contingent on local site characteristics and history. Most relevant to our work, prior cultivation could result in a homogenized microbial community that is resistant to changes in plant composition and other perturbations (Jangid et al., 2010, 2011). Homogenization of the microbial community with cultivation could result from the presence of crop monocultures or reduced soil structure and thus spatial heterogeneity from tilling, while microbial stability could be selected-for through repeated disturbance of the soil (reviewed in Griffiths and Philippot, 2013). In addition to experiencing strong seasonality, California grasslands frequently have well buffered soils, and many areas that undergo restoration have historically been cultivated (Stromberg et al., 2007). Soils at our site, for instance, were cultivated and then left fallow ca.10 years prior to treatment establishment, which may have contributed to the apparent insensitivity of the microbial community to further perturbation (Buckley and Schmidt, 2001). However, this is not always the case (Maul and Drinkwater, 2010). Steenwerth et al. (2005), for example, found that microbial communities in agricultural soils were less resistant to simulated rainfall than those in relict grasslands. The degree to which cultivation history moderates microbial stability to global and regional environmental changes is therefore an important area of research for future studies.

The extent to which a plant species affects soil microbial structure may be related to how long that species has been established at a given site (Batten et al., 2006; Kumschick et al., 2015). In our experiment, native and invaded treatments were markedly different in composition for 5 years, with native plots dominated by native plants and invaded plots dominated by exotic plants (Table S1 in Supplementary Material). However, it is possible that more time is required for detectable differences in soil microbial communities to develop between these plant communities. For example, Potthoff et al. (2006) suggested that 4 years may be insufficient for soil microbial communities to respond to the establishment of restored perennial grasslands. The relative abundance of a plant species within the community may further modify its effects on soil microorganisms (Hawkes et al., 2005; Elergsma and Ehrenfeld, 2011; Thomsen et al., 2011). While invaded and native plant communities were compositionally distinct for 5 years leading up to our sampling campaign, during the year we sampled there were more moderate differences in plant cover between the native and invaded communities (Table S2 in Supplementary Material). Thus, another possibility is that the soil microbial community responded so quickly to the convergence of plant communities in 2013 that any previous differences were no longer apparent. Finally, microbial communities can initially respond to changes in plant composition but revert back to an initial state over time (Lankau, 2011), making compositional resilience another potential explanation for why microbial communities were similar between native and invaded treatments.

If and how microbial community structure responds to changes in biotic and abiotic factors (e.g., plant composition and N supply) may also vary within a year (Docherty et al., 2012; Frenk et al., 2014). We sampled around the time of peak plant growth, and so did not capture seasonal patterns in microbial structure among the five treatments. Within a given growing season, plant species influence the soil environment through different mechanisms; for instance, in a California grassland potential rates of net N mineralization were found to correlate with bioavailable C in the fall and litter chemistry in the winter and spring (Eviner et al., 2006). Moreover, at times when decomposition of aboveground litter is high (for instance during winter in California, Eviner and Firestone, 2007), vegetation clipping and removal may have a pronounced effect on C supply to microorganisms (Wan and Luo, 2003). While it is thus possible that microbial communities may respond more strongly to manipulations at different periods in time, prior work documenting basic soil characteristics (pH, soil moisture, temperature) and N dynamics at our site shows very little interaction between the effects of treatment and time of sampling (Carey, 2014). Based on this, our results could be illustrative of a more enduring trend, although repeated sampling would be required to confirm this.

Overall, we demonstrate that key soil variables and potential rates of nitrification were altered by simulated environmental change in a semi-arid Mediterranean-type grassland. These changes did not result in distinct microbial communities, as determined by analysis of 16S rRNA genes. Seasonal and interannual variability in soil abiotic properties and plant community composition of Mediterranean sites, along with prior soil cultivation, could be responsible for promoting compositional stability, such that microorganisms in even the most altered soils (e.g., three simultaneous environmental changes) were able to persist without change. With global and regional change predicted to intensify (Sala et al., 2000), future work should focus on identifying critical thresholds of exotic plant abundance, frequency of vegetation removal, and rates of N deposition, beyond which microbial community composition and activity is altered in this and other systems. Identifying these critical levels of intensity in ecosystems with contrasting levels of seasonality, and that differ in site history, will be important for determining how ecosystems will respond to future environmental change.

### Conflict of interest statement

The authors declare that the research was conducted in the absence of any commercial or financial relationships that could be construed as a potential conflict of interest.
