# Supplementary material for: Soil microbial community structure is unaltered by plant invasion, vegetation clipping, and nitrogen fertilization in experimental semi-arid grasslands
Source: Front Microbiol. 2015 May 20;6:466. doi: 10.3389/fmicb.2015.00466 (PMC4438599; doi:10.3389/fmicb.2015.00466)

Figure S1. Boxplot of unweighted and weighted UniFrac distances within and between treatments.
Both = plots that were both clipped and fertilized. Top row = unweighted; bottom row = weighted.

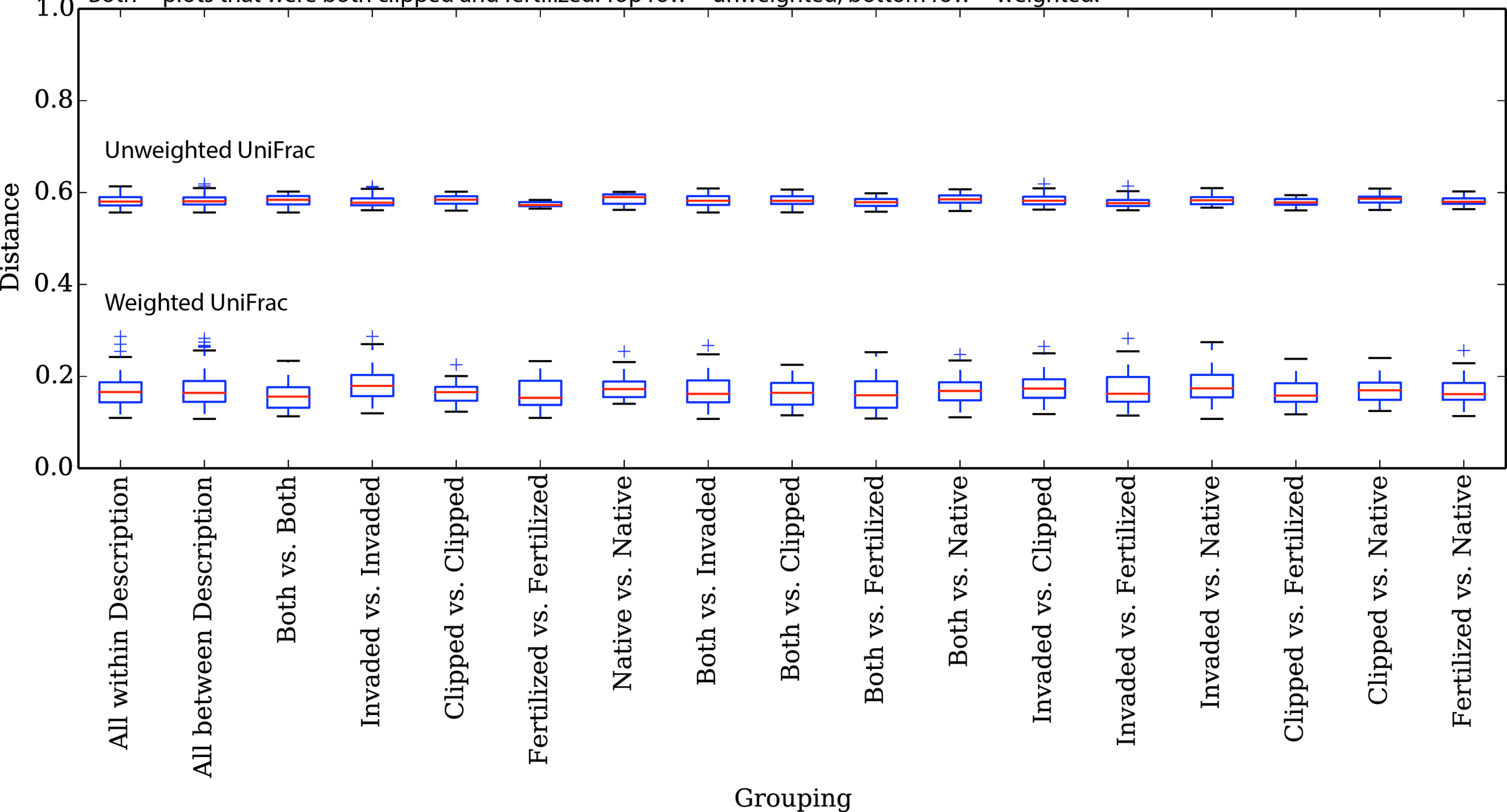

Supplement: Supplementary file 1 [file Image1.PDF]
