# Supplementary material for: Soil microbial community structure is unaltered by plant invasion, vegetation clipping, and nitrogen fertilization in experimental semi-arid grasslands
Source: Front Microbiol. 2015 May 20;6:466. doi: 10.3389/fmicb.2015.00466 (PMC4438599; doi:10.3389/fmicb.2015.00466)
Supplement: Supplementary file 2 [file Image2.PDF]

Supplementary Figure S2. PCoA of unweighted UniFrac metric

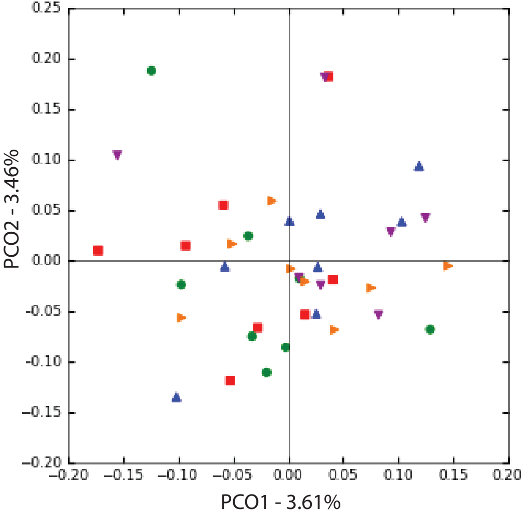

Legend:
Native = Purple triangle; Invaded = Green circle; Clipped = Blue triangle;
Fertilized = Yellow triangle; Clipped + Fertilized = Red square
