# Supplementary figures and images for: Soil microbial community structure is unaltered by plant invasion, vegetation clipping, and nitrogen fertilization in experimental semi-arid grasslands
Source: Front Microbiol. 2015 May 20;6:466. doi: 10.3389/fmicb.2015.00466 (PMC4438599; doi:10.3389/fmicb.2015.00466)

Figure S3. Rank abundance curve. Mean proportional abundance of each phylum by treatment.

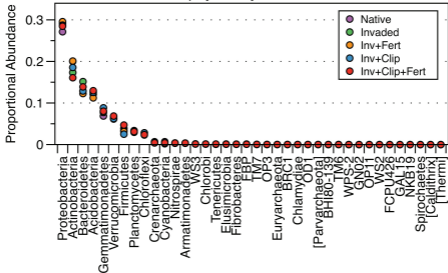

Supplement: Supplementary file 3 [file Image3.PDF]
