# Supplementary material for: Soil microbial community structure is unaltered by plant invasion, vegetation clipping, and nitrogen fertilization in experimental semi-arid grasslands
Source: Front Microbiol. 2015 May 20;6:466. doi: 10.3389/fmicb.2015.00466 (PMC4438599; doi:10.3389/fmicb.2015.00466)
Supplement: Supplementary file 4 [file Table1.PDF]

Supplementary Table S1: Percent cover of native, naturalized, and invasive species for native and invaded treatments from 2008 – 2013. Percentages for each plant group are averaged from the eight replicates per treatment.

		Spring 2008				Spring 2009				Spring 2010		
		% Native	% Naturalized	% Invasive		% Native	% Naturalized	% Invasive		% Native	% Naturalized	% Invasive
Native		100	0	0		100	0	0		95	0	2
Invaded		9	81	10		14	41	45		28	22	43
		Spring 2011				Spring 2012				Spring 2013		
		% Native	% Naturalized	% Invasive		% Native	% Naturalized	% Invasive		% Native	% Naturalized	% Invasive
Native		90	2	5		69	11	5		43	34	4
Invaded		22	8	70		29	10	45		34	22	9
