# Supplementary material for: Soil microbial community structure is unaltered by plant invasion, vegetation clipping, and nitrogen fertilization in experimental semi-arid grasslands
Source: Front Microbiol. 2015 May 20;6:466. doi: 10.3389/fmicb.2015.00466 (PMC4438599; doi:10.3389/fmicb.2015.00466)
Supplement: Supplementary file 5 [file Table2.PDF]

Supplementary Table 2: Percent cover of each native, naturalized, and invasive species across native and invaded treatments in 2013. Percentages for each species are averaged from the eight replicates per treatment. Other denotes the percent cover of non-planted species.

		Treatment	
Species	Plant Type	Native (%)	Invaded (%)
Bromus carinatus	Native	5.0	0.3
Elymus glaucus	Native	17.8	19.7
Elymus triticoides	Native	0.3	0.3
Stipa pulchra	Native	5.3	0.0
Poa secunda	Native	0.0	0.0
Festuca microstachys	Native	0.0	0.0
Acmispon americanus	Native	0.0	0.0
Lupinus bicolor	Native	0.3	0.0
Avena fatua	Naturalized	13.4	7.2
Festuca perennis	Naturalized	0.3	0.6
Bromus hordeaceus	Naturalized	7.8	5.6
Trifolium subterraneum	Naturalized	0.0	0.0
Aegilops triuncialis	Invasive	0.0	0.0
Elymus caput-medusae	Invasive	3.4	5.3
Other	---	20.6	34.1
