# Supplementary material for: Soil microbial community structure is unaltered by plant invasion, vegetation clipping, and nitrogen fertilization in experimental semi-arid grasslands
Source: Front Microbiol. 2015 May 20;6:466. doi: 10.3389/fmicb.2015.00466 (PMC4438599; doi:10.3389/fmicb.2015.00466)
Supplement: Supplementary file 6 [file Table3.PDF]

Supplementary Table 3: Main and interactive treatment effects of invasion, clipping, and N fertilization on ion-exchange resin NH_4^+ and NO_3^- ("fluxes"), nitrification potential, and denitrification potential.

Treatment	Flux Inorganic NH_4^+ (mg N kg^{-1} resin d^{-1})	Flux Inorganic NO_3^- (mg N kg^{-1} resin d^{-1})	Nitrification Potential (mg N kg^{-1} soil d^{-1})	Denitrification Potential (mg N_2O-N kg^{-1} soil h^{-1})
Invasion	0.81	0.18	**0.07**	0.33
Clipping	0.98	**0.06**	**0.10**	0.56
Fertilization	**0.08**	**0.00**	**0.05**	0.81
Clipping x Fertilization	**0.01**	0.92	0.24	0.47

P-values associated with invasion were generated by comparing native and invaded communities using a one-way ANOVA, and P-values for clipping and fertilization were generated using a two-way ANOVA. Significant ($P < 0.05$) and nearly significant ($P < 0.1$) values are highlighted in bold.
